# Study of Bulk Properties of Strange Particles in Au+Au Collisions at $\sqrt{s_{{}_{\mathrm{NN}}}}$ = 54.4 GeV

**DOI:** 10.3390/e24121720

**Published:** 2022-11-24

**Authors:** Li-Li Li, Abd Al Karim Haj Ismail

**Affiliations:** 1 Department of Basic Sciences, Shanxi Agriculture University, Jinzhong 030801, China; 2 College of Humanities and Sciences, Ajman University, Ajman P.O. Box 346, United Arab Emirates; 3 Nonlinear Dynamics Research Center (NDRC), Ajman University, Ajman P.O. Box 346, United Arab Emirates

**Keywords:** transverse momentum spectra, effective temperature, kinetic freeze-out temperature, transverse flow velocity, entropy parameter

## Abstract

We analyzed the transverse momentum $p_{T}$ spectra of various strange hadrons $K_{S}^{0}$, Λ($\bar{\Lambda }$) and $\Xi^{-}$(${\bar{\Xi }}^{+}$) at mid-rapidity (*y*) in different centrality intervals from Au+Au collisions at $\sqrt{s_{{}_{\mathrm{NN}}}}$= 54.4 GeV. The $p_{T}$ spectra of these strange hadrons are investigated by the Tsallis-like distribution, which satisfactorily fits the experimental data. The bulk properties of the medium produced in ultra-relativistic heavy-ion collisions at the kinetic freeze-out are reflected by measuring the hadron spectra. The effective temperature *T*, transverse flow velocity $\beta_{T}$, and mean $p_{T}$ along with other parameters that are strongly dependent on centrality and particle specie are extracted. The effective temperature of multi-strange particle ($\Xi^{-}$(${\bar{\Xi }}^{+}$)) is larger as compared to singly-strange particles Λ($\bar{\Lambda }$) and $K_{S}^{0}$. Furthermore, the kinetic freeze-out temperature *T*, transverse flow velocity $\beta_{T}$. and mean $p_{T}$ ($\langle p_{T}\rangle$) show a decreasing trend towards lower centrality, while the entropy parameter *q* increases from central to peripheral collisions. In addition, a positive correlation of $\langle p_{T}\rangle$ and *T* and a negative correlation of *q* and *T* are also reported.

## 1. Introduction

The quantum chromodynamics (QCD) calculations suggest that a deconfined phase of quarks and gluons is expected at a very high temperature or/and high baryon density [1,2,3,4,5,6]. A continuous and smooth crossover are predicted from a deconfined state of nuclear matter, and from the Quark-Gluon Plasma (QGP) to hadron gas phase state at low baryon chemical potential ($\mu_{B}$) and high temperature (*T*) [7]. At low *T* and high $\mu_{B}$ regions, a first order phase transition is also predicted by the theoretical calculations, which may end at the possible QCD critical point [8,9,10,11,12]. The mapping of the QCD phase diagram is one of the main objectives of the Relativistic Heavy Ion Collider (RHIC) Beam Energy Scan (BES) program. Ultra-relativistic heavy-ion collisions are considered as the most promising tool to study the deconfined state of nuclear matter, and the QGP where the partonic interactions dominate. The space-time evolution of various colliding systems is a complex process in which different degrees of freedom are involved under different spatio-temporal coordinates. This complexity makes it difficult to use a theory in order to describe and understand the development of the entire system. In heavy-ion collisions, a thermalized system is produced, where *T* and $\mu_{B}$ can be varied by changing the collision energies. Therefore, in order to study and understand the possible signatures of phase boundary of the QCD matter, RHIC carried out the first phase of the BES program in 2010 and 2011 [13,14,15,16,17,18] by varying the colliding energies. In the year 2017, the STAR experiment at RHIC collected high statistics data from Au+Au collisions at $\sqrt{s_{{}_{\mathrm{NN}}}}$ = 54.4 GeV, which allowed us to extend the precise measurements of various parameters, especially from intermediate to high $p_{T}$ regions [18].

It is equally important to study the invariant yield and transverse momentum ($p_{T}$) spectra of various identified and strange particles, which provide a baseline to understand the freeze-out properties of the system produced in heavy-ion collisions. In high energy heavy-ion collision experiments, two freeze-out stages can be observed; the chemical freeze-out and kinetic freeze-out stages. In the chemical freeze-out stage, the inelastic collisions between the particles ceases, no new particles are further produced, and the yields of particle type becomes fixed. Many of the available thermodynamics models are used to extract the baryon chemical potential and chemical freeze-out temperature at this stage [19,20,21,22]. After chemical freeze-out, only elastic collision is involved in particle collisions. At the kinetic freeze-out stage and with further expansion of the system, the separation between the particles becomes large, and the elastic collision among particles ceases. After this stage, the momenta of particles also becomes fixed, and particles start propagating towards the detector. The $p_{T}$ spectra of particles are helpful to study the information of the kinetic freeze-out stage. Various hydro-dynamical models are used to extract the kinetic freeze-out temperature ($T_{k}$ or $T_{0}$) and radial flow velocity (β), which in fact carry the information of transverse expansion of the system [19,20,23,24].

The study of the kinetic freeze-out stage is very complex, and important single, double, triple, and differential (multiple) freeze-out scenarios have been reported in different studies [25,26,27,28,29,30,31]. Additionally, the freeze-out temperature $T_{0}$ is observed to show an increasing trend with colliding energy up to 10 GeV as well as centrality, which is also very complex [32,33,34,35]. The increasing trend of $T_{0}$ with collision energy becomes indefinitely saturated, which could be due to the flow effects. On the contrary, the contributions of flow effects and thermal motion are taken into account in the effective temperature *T*, which has definite trend and is different from $T_{0}$. The dependence of kinetic freeze-out temperature on centrality is also very complex, and has shown different behavior in different literature, i.e., an increasing trend of kinetic freeze-out temperature from central to peripheral collisions [36], a decreasing trend from central to peripheral collisions [34,37,38,39], and invariant from central to peripheral collision [40].

The $p_{T}$ spectra of hadrons are important tools to understand the dynamics of the particles production in high energy collisions, and we can extract the freeze-out parameters such as chemical freeze-out temperature ($T_{ch}$), kinetic freeze-out temperature ($T_{0}$), effective temperature (*T*), transverse flow velocity ($\beta_{T}$), and kinetic freeze-out volume (*V*) from the $p_{T}$ spectra of the particles by using different hydro-dynamical models such as Blast-Wave model with Boltzmann Gibbs statistics (BGBW) [24,41,42], blast-wave model with Tsallis statistics (TBW) [25], Hagedorn thermal model [43], Standard distribution [44], modified Hagedron model [45], and other thermodynamic models [46,47,48,49,50]. Different models have different limitations, for instance, BGBW, Standard distribution, and Hagedorn thermal model are valid for the narrow $p_{T}$ region from 2–3 GeV/c, while TBW model and Tsallis-like distribution can cover a wide $p_{T}$ range. Moreover, TBW or other models that cover the wide $p_{T}$ range fail to cover the very narrow $p_{T}$ range ($p_{T}$ < 0.5 GeV/c), which is responsible for the resonance production, where a Tsallis-like function can cover it successfully.

In the present work, Tsallis-like distribution is used to analyze the $p_{T}$ spectra of various strange hadrons, and extract the effective temperature (*T*), the entropy index (*q*), mean transverse momentum ($\langle p_{T}\rangle$), the kinetic freeze-out temperature ($T_{0}$), and the transverse flow velocity ($\beta_{T}$). It is very important to extract these parameters and study their correlation because they provide crucial information about the final state particles. In addition, such studies are considered as important tools to understand the dynamics of the particles production in high energy collisions, and they are very useful in understanding the microscopic features of degrees of equilibrium and their dependencies on the number of participants in the system. Besides, such studies may also provide some useful information about the formation of super hadronic dense matter. The results are then verified by studying the $p_{T}$ spectra of various strange hadrons, $K_{S}^{0}$, Λ($\bar{\Lambda }$) and $\Xi^{-}$(${\bar{\Xi }}^{+}$) produced from Au+Au collisions at $\sqrt{s_{{}_{\mathrm{NN}}}}$ = 54.4 GeV by the STAR experiment at RHIC [18]. The rest of the paper is organized as follows: the method and formalism are briefly discussed in Section 2, the results are discussed in Section 3, and the summary and conclusion are described in Section 4.

## 2. Methods and Models

The Tsallis-like function describes very well the very narrow, narrow, wide, and very wide $p_{T}$ distributions of hadrons in high-energy collisions. These regions are discussed in detail in Ref. [40]. There are various forms and revised versions of the Tsallis distribution and the Tsallis-like distribution in terms of rapidity. A simple form of the function of *y* and $p_{T}$ at mid-rapidity is given as follows [44,51,52,53]:

(1) $$\frac{d^{2}N}{dydp_{T}}\propto \frac{dN}{dy}m_{T}{\left[1+\frac{\left(q-1\right)\left(m_{T}-\mu -m_{0}\right)}{T}\right]}^{-q/(q-1)},$$

where *N* represents the number of particles, $m_{T}$ is the transverse mass and can be written as $m_{T}=\sqrt{p_{T}^{2}+m_{0}^{2}}$, which can be obtained using $p_{T}$, and $m_{0}$ is the rest mass of hadron. *q* is the entropy parameter that reflects the degree of non-equilibrium of the system. In general, q=1 is correspondent to an ideal equilibrium state while when *q* is much larger than unity (q>>1), the system is at a non-equilibrium state. μ is the chemical potential that refers to the degree of imbalance between matter and anti-matter, and can be given as:

(2) $$\mu_{b}=\frac{1.3075}{1+0.288\sqrt{{}_{NN}}}.$$

Equation (2) is the joint density function and is normalized to 1, and it can be transformed to the probability function of *y* and $p_{T}$, respectively. Therefore, Equation (1) is revised as:

(3) $$f\left(p_{T},T\right)=\frac{1}{N}\frac{dN}{dp_{T}}=Cm_{T}{\left[1+\frac{\left(q-1\right)\left(m_{T}-\mu -m_{0}\right)}{T}\right]}^{-q/(q-1)}.$$

For further details of the whole phenomenology of the model in case of double or even multiple components, one can consult Refs. [33,54,55]. It should be noted that the term $m_{T}$-μ-$m_{0}$ can also be written as $m_{T}$-μ. They are essentially the same because $m_{0}$ can fit into μ. It is just the matter of magnitude.

## 3. Results and Discussion

**Figure 1:**
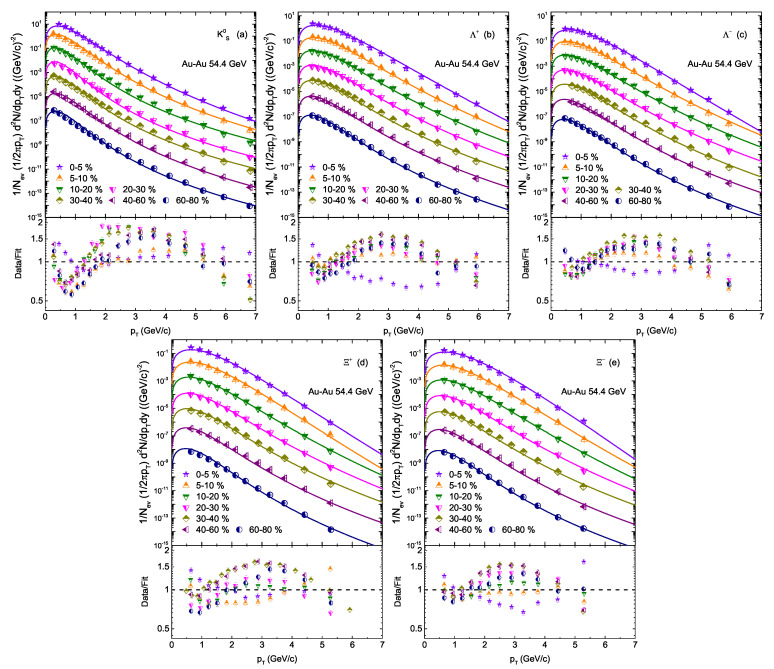
Transverse momentum $p_{T}$ spectra of $K_{S}^{0}$, Λ($\bar{\Lambda }$) and ${\bar{\Xi }}^{+}$($\Xi^{-}$) in Au+Au collisions at $\sqrt{s}$ = 54.4 GeV in different centrality bins from the STAR experiment [18] fitted with Tsallis-like distribution. Data/fit ratio is also shown at the lower panel.

Figure 1 presents the transverse momentum ($p_{T}$) spectra of $K_{S}^{0}$, Λ($\bar{\Lambda }$) and $\Xi^{-}$(${\bar{\Xi }}^{+}$) in Au+Au collisions at $\sqrt{s_{{}_{\mathrm{NN}}}}$ = 54.4 GeV, which is analyzed with Tsallis-like distribution. The analysis is performed for different centrality bins at mid-rapidity (|y|<0.5). The experimental data is measured by the STAR experiment [18] and shown using markers in different colors, while the results of the Tsallis-like distribution are presented by the curves to the experimental data. The ratio of the experimental data to the model fit are shown in the lower panels of the figure. The extracted parameters and respective $\chi^{2}/dof$ are listed in Table 1. It can be observed that the experimental data is well described by Equation (3).

The least square method is used to extract the best parameters by fitting the spectra. The statistical and systematic errors are added up in quadrature for the error calculations in $\chi^{2}$ and the parameters, which minimize the $\chi^{2}$, are chosen as the best parameters.

We would like to point out that the fit results in some cases for a few points deviates from the data, as shown in the lower panel of Figure 1. Normally, if it deviates from 0.5 to 1.5, it is considered to be normal. In the present study, most of the points are in the range of 0.5 to 1.5 in data/fit ratio except for few cases in the $p_{T}$ range of (2−4GeV/c), and that results in high $chi^{2}/$ dof shown in Table 1. This deviation has been considered to be large by one of the respected anonymous referees. However, we believe that such a deviation is due to the fact that, in the present work, we have used a single component function of Tsallis-like distribution. If we would have used a two or multi-component function, the situation would be different, but the second or multi- component from the high $p_{T}$ region slightly contributes to the derived parameters. Therefore, it was not considered in the present work.

**Figure 2:**
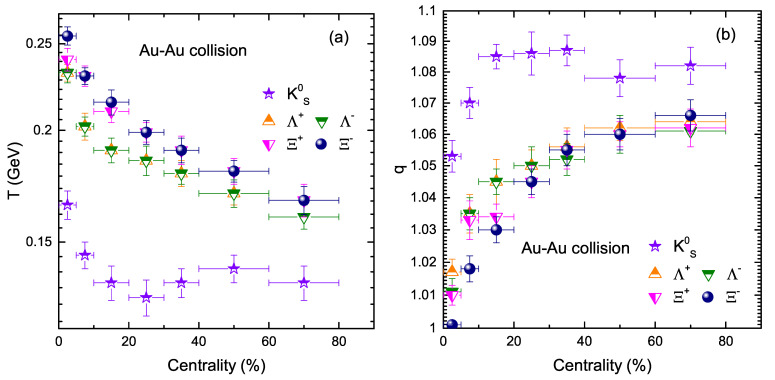
Left: (**a**) Effective temperature *T* as a function of centrality. Right: (**b**) Entropy index *q* as a function of centrality.

In order to check the trend of parameters in respect to centrality, we present Figure 2a, which shows the effective temperature as a function of different centrality classes, where a strong centrality dependence of effective temperature *T* is observed. This behavior shows that a large amount of energy is transmitted to the interacting system due to the barbaric reaction in central collisions systems, which decreases towards lower centrality classes. It can also be observed that *T* values are larger in the case of a heavier particle, i.e., *T* (Ξ) > *T* (Λ) > *T* ($K_{S}^{0}$) for all centrality bins. However, in comparison to other strange particles, $K_{S}^{0}$, as a lighter particle, shows non-monotonic centrality dependence and the value of *T* is smaller. In addition, *T* is seen to be higher with the increase in strangeness content, indicating that multi-strange particles freeze-out earlier. The degree of non-extensiveness of the system is reflected by the entropy index *q*, presented in Figure 1b. When q=1, it means that the system is in equilibrium state, while a non-equilibrium state of the interacting system happens when q>1. Figure 1b shows the centrality dependence of entropy index *q* for $K_{S}^{0}$, Λ($\bar{\Lambda }$) and $\Xi^{-}$(${\bar{\Xi }}^{+}$) in Au+Au collisions at $\sqrt{s_{{}_{\mathrm{NN}}}}$ = 54.4 GeV. It can be seen that the *q* value increases from central to peripheral collisions for all strange particles. The *q*-value of Λ($\bar{\Lambda }$) and $\Xi^{-}$(${\bar{\Xi }}^{+}$) in central collisions is close to unity, which indicates a rapid approach of the system to equilibrium, while towards periphery, the system deviates far from a state of equilibrium. However, no strong centrality dependence is observed in case of $K_{S}^{0}$*q*-value and it is almost independent of centrality. However, the *q*-value for $K_{S}^{0}$ is observed to be higher as compared to other particles under study, which indicates that $K_{S}^{0}$ hardly interacts with the created medium in the collision and shows a minimal tendency for equilibrium.

Furthermore, in order to study the centrality dependence on transverse momentum, the mean transverse momentum ($\langle p_{T}\rangle$) is calculated using the probability density function:

(4) $$<p_{T}>=\int_{0}^{\infty }p_{T}\times f\left(p_{T}\right)dp_{T}dy.$$

**Figure 3:**
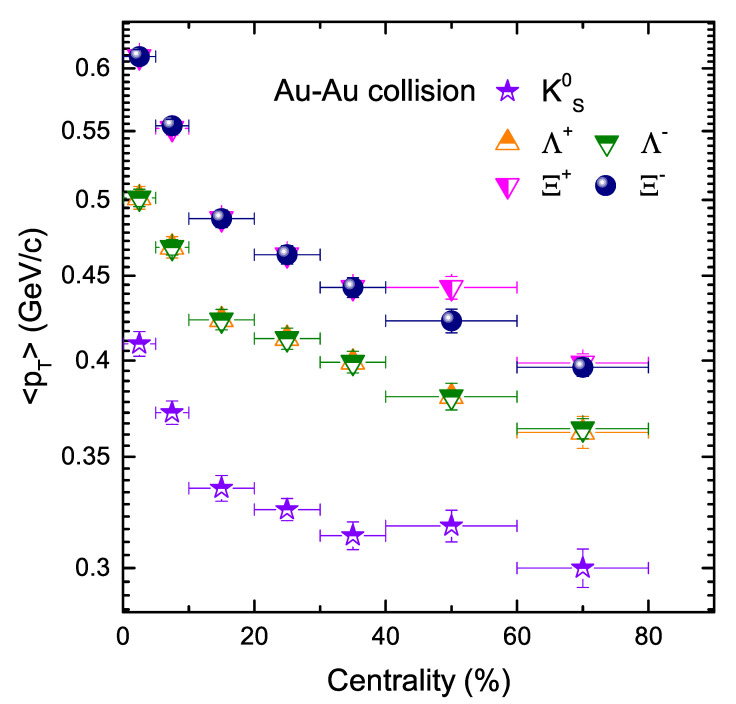
Comparison of transverse momentum ($\langle p_{T}\rangle$) as a function of centrality of different strange hadrons.

In Figure 3, we have shown the effect of centrality on mean transverse momentum ($\langle p_{T}\rangle$) of various strange particles in Au+Au collisions. It has been observed that $\langle p_{T}\rangle$ shows a decreasing trend from central to peripheral collisions. This can be explained by the fact that a larger momentum is gained by the system in central collisions as compared to peripheral collisions, which results in further multiple scattering in central collisions and which tends to decrease with decreasing centrality. It can also be explained that the effect of flow decreases towards periphery, which explains why $\langle p_{T}\rangle$ decreases towards periphery. In addition, $\langle p_{T}\rangle$ is also dependent on $m_{0}$. Larger $\langle p_{T}\rangle$ corresponds for the heavier particles, which evince that the radial flow is larger for the heavier particles.

**Figure 4:**
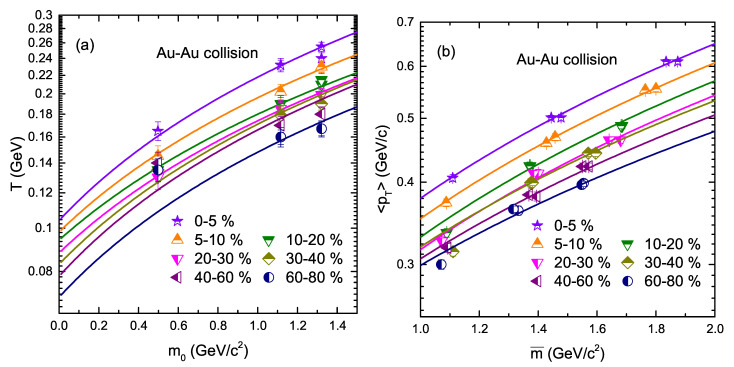
(**a**) Effective temperature *T* as a function of mass ($m_{0}$) (**b**) $\langle p_{T}\rangle$ as a function of $\bar{m}$.

Since the effective temperature displays the contributions of both the thermal motion and flow effect [56], the former describes the kinetic freeze-out temperature while the latter shows the flow effect. In order to get the values of freeze-out temperature $T_{0}$ and $\beta_{T}$, we have analyzed the values of effective temperature *T* presented in Table 1, and calculated $\langle p_{T}\rangle$ and mean moving mass ($\bar{m}$). The isotropic assumption in the rest frame of emission is interpreted in the calculation from $p_{T}$ to $\langle p_{T}\rangle$ and $\bar{m}$ [37,57,58,59,60,61] by using the Monte Carlo method. The relation of *T* and $m_{0}$, and $\langle p_{T}\rangle$ and $\bar{m}$ are displayed in Figure 4. Fit values are obtained according to the least square method. According to Refs. [24,62,63,64], the intercept between *T* and $m_{0}$ is regarded as the kinetic freeze-out temperature while the slope between $\langle p_{T}\rangle$ and $\bar{m}$ is regarded as the transverse flow velocity. The slope and intercept extracted from the linear fitting to *T* and $m_{0}$, $\langle p_{T}\rangle$ and $\bar{m}$ in Figure 4a,b are listed in Table 2 and Table 3, respectively.

**Figure 5:**
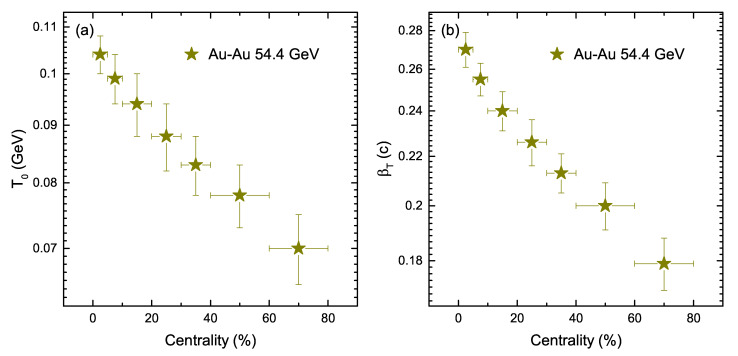
(**a**) Kinetic freeze-out temperature $T_{0}$, (**b**) transverse flow velocity ($\beta_{T}$) as a function of centrality of different strange hadrons in Au+Au collisions at $\sqrt{s_{{}_{\mathrm{NN}}}}$ = 54.4 GeV.

Figure 5a shows the extracted kinetic freeze-out temperature $T_{0}$ as a function of centrality. It can be seen that the $T_{0}$ tends to decrease non-monotonically from central to peripheral collisions. In central collisions, this non-monotonic decrease indicates that more participant nucleons are interacting as compared to peripheral collisions, which naturally results in a higher degree of excitation in central collisions compared to peripheral collisions. Figure 5b shows $\beta_{T}$ as a function of centrality. The value of $\beta_{T}$ is observed to be larger in most central collisions as compared to peripheral collisions. This can be explained by the presence of more violent reaction due to the high intensity squeeze, in which a larger pressure gradient is produced in central collisions, and that results in the rapid expansion of the system. On the other hand, $\beta_{T}$ becomes smaller towards the periphery because of less violent reactions and less of a pressure gradient.

**Figure 6:**
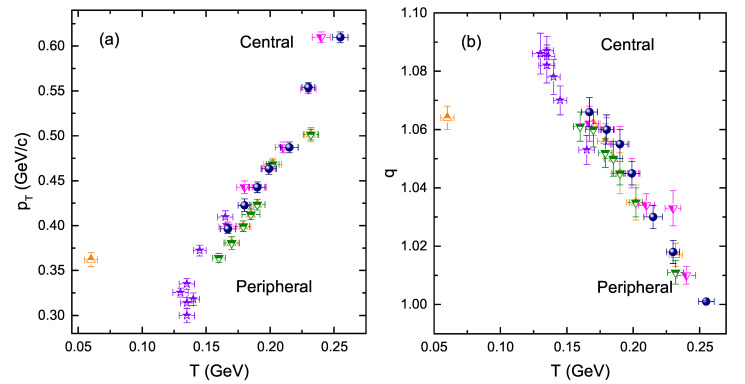
(**a**) Correlation of $\langle p_{T}\rangle$ and *T*, (**b**) correlation of *q* and *T*, shown for different strange particles $K_{S}^{0}$, Λ($\bar{\Lambda }$) and ${\bar{\Xi }}^{+}$($\Xi^{-}$) in Au+Au collisions at $\sqrt{s}$ = 54.4 GeV.

Figure 6 shows the correlations of the parameters with each other. In the left, Figure 6a shows the correlation of $\langle p_{T}\rangle$ and *T* while Figure 6b shows the correlation of *q* and *T*. Each data point in panel (a) and (b) show the correlation of $p_{T}$ and *T*, and *q* and *T* in each centrality bin, respectively. The positive correlation among $\langle p_{T}\rangle$ and *T* is reported, which evinces that the larger $<p_{T}>$ (energy) is transferred to the system due to the very intense collision. This corresponds to a higher degree of excitation and results in a larger $T_{0}$. However, Figure 6b reported a negative correlation among *q* and *T*. Larger *T* for heavier particles and in central collisions refers to the quick approach of equilibrium of the system. On the other hand, smaller *q* refers to the state that is close to equilibrium. Hence, *q* is smaller for heavier particles and in central collisions system, and *T* is larger for heavier particles in central collisions.

During the fitting procedure, we imposed some physical restrictions on the parameters. For instance, *T* for $K_{S}^{0}$ is allowed to vary between 0.165 to 0.135 GeV, while *q* is restricted from 1.063 to 1.082. This is similar to other particles. Larger *T* in the central collision refers to higher pressure gradients in the collision zone, while smaller *q* in the central collision zone shows the quick approach of the system in reaching the equilibrium state. We know that the main purpose of the high energy experiments is to identify and study the physical aspects of QCD at the non-perturbative regime, as well as the running coupling and the structure of the QGP. In this context, the present results propose the earlier freeze-out of massive particles rather than the lighter ones, which are closer to equilibrium. In addition, all freeze-out of the studied particles in the central collision systems are closer to equilibrium.

Before presenting the conclusions, we would like to explain that the extraction of the parameters from the $p_{T}$ spectra of hadrons is very valuable. The extraction of temperature and flow of the particles describe the thermal excitation degree of the system, and the radial flow exhibits the collective motion. The present work studied the effective temperature, which is a combination of the kinetic freeze-out temperature (the final state temperature of the particles) and radial flow. The final state temperature of hadrons is usually called the kinetic or thermal freeze-out temperature, which refers to the temperature of the emission when the inelastic collisions cease and there are only elastic collisions among the particles. One other thing we would like to highlight is that the QGP matter is already observed in different experiments at RHIC and LHC, but its direct detection is still pending due to the reason that the life time of the QGP is too short, and a lot of valuable information is lost. The transverse momentum spectra of hadrons can be used to extract the lost information such as transverse excitation and dynamic expansion of the collision system.

Furthermore, the transverse momentum distribution of the final state hadrons can reflect the information of the transverse excitation degree and dynamic expansion of the system. If the hot and dense matter produced during the high energy collisions is assumed to be a huge fireball, ingredients such as quarks and gluons, which build up this fireball, can be interpreted as a source of emission, and the transverse momentum ($p_{T}$) spectra of the final state hadrons are given by these sources. The transverse expansion of the reaction system is caused by a transverse pressure gradient, especially at the edges. Transverse expansion is very sensitive to the state of matter, therefore, during the phase transition, the pressure of the system remains basically the same but the energy density changes a lot.

## 4. Conclusions

In this paper, we studied the bulk properties of various strange hadrons $K_{S}^{0}$, Λ($\bar{\Lambda }$) and $\Xi^{-}$(${\bar{\Xi }}^{+}$) in Au+Au collisions at $\sqrt{s_{{}_{\mathrm{NN}}}}$ = 54.4 GeV measured by the STAR experiment at RHIC by using the Tsallis-like distribution. In order to completely understand the matter formed in these collisions, studying the microscopic properties of the particles is equally important. The main findings and conclusions of the study are summarized as follows:

- The transverse momentum $p_{T}$ spectra of various strange hadrons $K_{S}^{0}$, Λ($\bar{\Lambda }$) and $\Xi^{-}$(${\bar{\Xi }}^{+}$) are analyzed at mid-rapidity (*y*) in different centrality intervals from Au+Au collisions at $\sqrt{s_{{}_{\mathrm{NN}}}}$ = 54.4 GeV. The Tsallis-like distribution is used to analyze the spectra of these strange hadrons. The model results showed an agreement with the experimental data from the STAR experiment;
- The centrality dependence of entropy index *q* is studied for $K_{S}^{0}$, Λ($\bar{\Lambda }$) and $\Xi^{-}$(${\bar{\Xi }}^{+}$) in Au+Au collisions. The *q*-value of Λ($\bar{\Lambda }$) and $\Xi^{-}$(${\bar{\Xi }}^{+}$) in central collisions is close to unity, which indicates a rapid approach of the system to equilibrium, while towards periphery, the system deviates far from the equilibrium state. On the other hand, $K_{S}^{0}$ does not show strong centrality dependence in *q*-value, indicating that it hardly interacts with the created medium during high energy heavy-ion collisions. Moreover, the mean $p_{T}$ ($\langle p_{T}\rangle$) is observed to show a decreasing trend from central to peripheral collisions indicating that, in central collisions, the amount of momentum gained by the system and multiple scatterings is larger in comparison to the peripheral collisions, and the radial flow is more pronounced;
- The effective temperature *T*, mean transverse momentum $\langle p_{T}\rangle$ and the entropy *q* are mass dependent and they increase with the mass of the particle. Furthermore, the isotropic assumption in the rest frame of emission is interpreted in the calculation from $p_{T}$ to $\langle p_{T}\rangle$ and $\bar{m}$ using the Monte-Carlo method;
- The freeze-out temperature $T_{0}$ and transverse velocity $\beta_{T}$ are extracted using an alternative method, and they are observed to be decreasing from the central to peripheral collisions. The reason for this behavior is that the degree of excitation of the system in central collisions is much higher than that of peripheral collisions. and it could also be due to the large number of participating nucleons that are experiencing a stronger squeeze as more violent reaction takes place and a high pressure gradient is produced. This results in a rapid expansion of the system in central collisions as compared to peripheral collisions;
- The correlation between mean transverse momentum $\langle p_{T}\rangle$ and effective temperature *T*, and entropy *q* and effective temperature *T*, are reported. The former correlation is positive while the latter correlation is negative, and they show the early equilibrium state of the central collisions as well as for massive particles;
- Finally, the presented results are consistent with our previous results in Ref. [65] with the BEAM energy Scan, in which we analyzed the light nuclei and obtained a decreasing trend of *T* as well as $p_{T}$ from central to peripheral collisions. However, there is an inconsistency in the present results with Ref. [36], where the light nuclei are analyzed using the Blast-Wave model. However, the same model also gives different results if we use different methods of extraction of the parameters, as well as if the limits and conditions applied to the model are different [20,42].

## Figures and Tables

**Table 1:** Spectrum, values of free parameters (*T* and *q*), normalization constant ($N_{0}$), $\chi^{2}$, and degree of freedom (dof) corresponding to the curves in Figure 1.

Collision	Particle	Centrality	*T* (GeV)	*q*	$N_{0}$	$\chi^{2}$/*dof*
Au-Au 54.4 GeV	$K_{0}^{S}$	0–5%	0.165±0.006	1.053±0.005	1480±84	111/16
		5–10%	0.145±0.005	1.070±0.005	300±33	30/17
		10–20%	0.135±0.006	1.085±0.004	24±1.4	110/17
		20–30%	0.130±0.006	1.086±0.007	1.6±0.06	44/17
		30–40%	0.135±0.005	1.087±0.005	0.1±0.03	139/17
		40–60%	0.140±0.006	1.078±0.006	0.004±0.0004	61/17
		60–80%	0.135±0.006	1.082±0.005	$1.3\times 10^{-4}\pm 4\times 10^{-5}$	58/17
Au-Au 54.4 GeV	$\Lambda^{+}$	0–5%	0.232±0.005	1.017±0.004	380±43	61/15
		5–10%	0.202±0.007	1.035±0.006	40±5	70/15
		10–20%	0.190±0.006	1.045±0.007	3.8±0.2	25/15
		20–30%	0.185±0.007	1.050±0.005	0.26±0.04	82/15
		30–40%	0.179±0.006	1.056±0.006	0.016±0.004	37/15
		40–60%	0.170±0.005	1.062±0.004	$9\times 10^{-4}\pm 5\times 10^{-5}$	43/15
		60-80%	0.160±0.005	1.064±0.004	$2.8\times 10^{-5}\pm 5\times 10^{-6}$	60/15
Au-Au 54.4 GeV	$\Lambda^{-}$	0–5%	0.232±0.006	1.011±0.004	164±13	25/15
		5–10%	0.202±0.005	1.035±0.005	20±1.6	28/15
		10–20%	0.190±0.006	1.045±0.004	1.7±0.04	98/15
		20–30%	0.185±0.007	1.050±0.005	0.11±0.04	95/15
		30–40%	0.179±0.005	1.052±0.005	0.008±0.0004	70/15
		40–60%	0.170±0.006	1.060±0.006	$5\times 10^{-4}\pm 4\times 10^{-5}$	32/15
		60–80%	0.160±0.005	1.061±0.005	$1.3\times 10^{-5}\pm 4\times 10^{-6}$	84/15
Au-Au 54.4 GeV	$\Xi^{+}$	0–5%	0.240±0.007	1.010±0.003	$8.8\times 10^{-4}\pm 4\times 10^{-5}$	31/10
		5–10%	0.230±0.006	1.033±0.006	5±0.3	18/10
		10–20%	0.210±0.006	1.034±0.004	0.4±0.03	87/10
		20–30%	0.199±0.005	1.045±0.005	0.027±0.005	122/10
		30–40%	0.190±0.007	1.055±0.006	0.002±0.0003	75/10
		40–60%	0.180±0.006	1.060±0.004	$8\times 10^{-5}\pm 6\times 10^{-6}$	82/10
		60-80%	0.167±0.007	1.062±0.006	$2.5\times 10^{-6}\pm 5\times 10^{-7}$	39/10
Au-Au 54.4 GeV	$\Xi^{-}$	0–5%	0.255±0.007	1.001±0.0003	27±2.7	28/10
		5–10%	0.230±0.005	1.018±0.004	3±0.4	55/10
		10–20%	0.215±0.007	1.030±0.004	3±0.3	93/10
		20–30%	0.199±0.006	1.045±0.004	0.018±0.003	39/10
		30–40%	0.190±0.006	1.055±0.005	0.0012±0.0003	68/10
		40–60%	0.180±0.005	1.060±0.005	$6\times 10^{-5}\pm 5\times 10^{-6}$	76/10
		60–80%	0.167±0.006	1.066±0.005	$1.8\times 10^{-6}\pm 4\times 10^{-7}$	46/10

**Table 2:** Values of intercepts, slopes, and $\chi^{2}$ in the linear fittings in Figure 4a.

Figure	Collision	Centrality	Intercept	Slope	$\chi^{2}$
Figure 4a	Au-Au	0–5%	0.104±0.005	0.114±0.006	4.5
		5–10%	0.098±0.006	0.098±0.008	1.5
		10–20%	0.094±0.006	0.094±0.006	1.3
		20–30%	0.088±0.005	0.086±0.006	0.3
		30–40%	0.083±0.006	0.088±0.006	5
		40–60%	0.078±0.005	0.088±0.005	16
		60–80%	0.070±0.005	0.085±0.006	20

**Table 3:** Values of intercepts, slopes, and $\chi^{2}$ in the linear fittings in Figure 4b.

Figure	Collision	Centrality	Intercept	Slope	$\chi^{2}$
Figure 4b	Au-Au	0–5%	0.107±0.006	0.271±0.009	4
		5–10%	0.097±0.005	0.255±0.010	2
		10–20%	0.090±0.005	0.240±0.009	16
		20–30%	0.090±0.008	0.226±0.008	10
		30–40%	0.106±0.009	0.213±0.011	34
		40–60%	0.106±0.008	0.200±0.0010	16
		60–80%	0.120±0.007	0.179±0.008	10

## Data Availability

Not applicable.
